# “Read” and ‘Tailor’ Your Treatment

**DOI:** 10.22038/ijorl.2019.29914.1972

**Published:** 2019-11

**Authors:** Kausalya K Sahu, Deviprasad Dosemane, Meera Khadilkar, Deeksha Shetty, Madhurya Raminen

**Affiliations:** 1 Department of Pathology, Kasturba Medical College, Mangalore, Manipal Academy of Higher Education, Mangalore, India.; 2 Department of Otorhinolaryngology, Kasturba Medical College, Mangalore, Manipal Academy of Higher Education, Mangalore, India.

**Keywords:** Ethmoidal polyps, Hamartoma, Nasal mass, Nasal polyps, Respiratory epithelial adenomatoid hamartoma (READ), Sinonasal tract

## Abstract

**Introduction:**

The sinonasal tract is an area that may be affected by various types of neoplastic lesions with more variety than what is encountered in other parts of the upper airway and food passage.

**Case Report:**

An elderly gentleman of 65 years complained of nose block on both sides for 3 months. On examination, he had firm polypoidal masses arising from the middle meatus and septum on both sides. Computed tomography scan of the sinuses revealed polypoidal mucosal thickening in bilateral sinuses with bilateral concha bullosa. The patient underwent functional endoscopic sinus surgery with the complete excision of the masses from the lateral wall and septum under general anesthesia. The results of the histopathological examination showed inflammatory changes in polypoidal tissues from the right maxillary, bilateral ethmoidal sinuses, and bilateral septal masses. The lesion in the left middle meatus showed the features of respiratory epithelial adenomatoid hamartoma (READ). The case had no evidence of residual or recurrent lesion during 6 months after the surgery.

**Conclusion:**

The READ is a rare lesion observed in the sinonasal tract. It is a diagnostic dilemma for clinicians as it can be misdiagnosed as neoplastic lesions, such as inverted papilloma or adenocarcinoma, which would warrant radical surgery or sinonasal polyposis and be treated inadequately.

## Introduction

The sinonasal tract is an area that may be affected by various types of neoplastic lesions with more variety than what is encountered in other parts of the upper airway and food passage. The common differential diagnosis of sinonasal mass includes benign lesions, such as sinonasal polyps (including ethmoidal and antrochoanal polyps), papillomas, haemangioma, gliomas, dermoids, inverted papillomas, and malignancies (1–3).

## Case Report

An elderly gentleman of 65 years complained of bilateral nasal block and hyposmia for 3 months. He had no previous complaints suggestive of long-standing sinusitis or allergy. There was no history of epistaxis, ocular symptoms or trauma. The patient was a known hypertensive using regular medications with no other comorbid illness. There was no history of nose surgical treatment or paranasal sinus diseases. 

No abnormality was observed in the results of routine laboratory investigations. Anterior rhinoscopy showed deviated nasal septum to the right side with polypoidal masses observed in the bilateral nasal cavities arising from middle meatus, and right-side mass was noticed extending inferiorly up to the inferior turbinate. Single sessile mass was observed attached to the septum on each side. The visible part of the mass was firm with smooth surface and nontender and did not bleed on touch on probe test. 

The resuls of computed tomography (CT) scan of the sinuses revealed polypoidal mucosal thickening in the bilateral middle meatus, sphenoid sinus, and left frontal sinus. There were bilateral conch a bull OSA, bilateral accessory maxillary ostia, and a globular polypoidal mass attached to the left middle turbinate lying in the middle meatus region (Fig.1).

 The patient was operated to achieve the complete excision of the masses from the lateral wall and septum under general anesthesia. 

During the surgery, the results of nasal endoscopy confirmed the anterior rhinoscopy findings (Fig. 2), and even the nasopharynx was normal. All the masses from both sides were removed in Toto and sent for histopathological examination. The globular polypoidal mass attached to left middle turbinate was measured at 3.5×2.5×0.7 cm^3^ with its base 1.5 cm in length (Fig.3). 

**Fig1:**
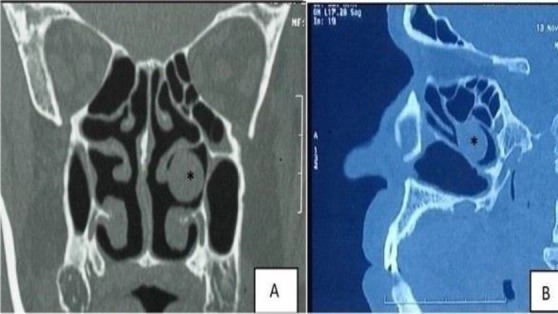
Computed tomography of osteomeatal complex; A) coronal section and B) sagittal section showing globular polypoidal mass attached to left middle turbinate lying in left middle meatus region

**Fig 1:**
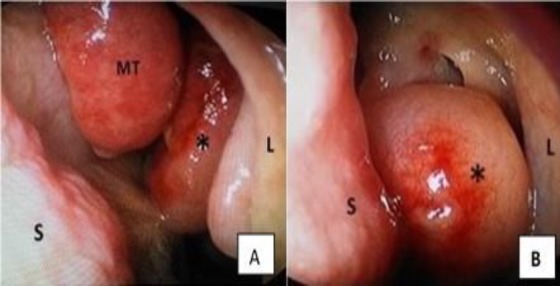
A and B – left side of nose, intraoperative findings S: Septum, M: Middle turbinate, L: Lateral wall of nose,*: Polypoidal mass observed in left middle meatus

**Fig 3:**
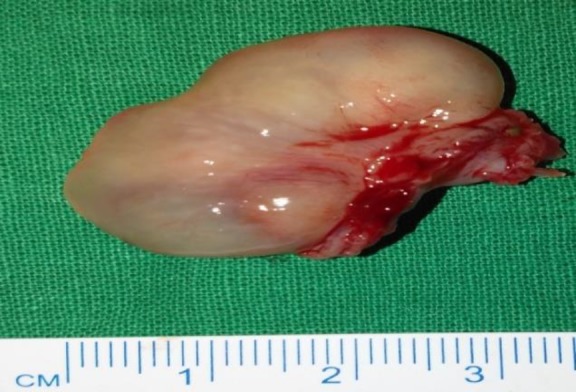
Gross specimen of mass excised from left middle meatus

On sectioning, the mass was pale brown to pale white in color and covered by mucosa. Cut section showed a cyst measured at 0.4 cm in diameter, along with five pale white circular lesions larger measured at 0.3 cm and smaller measured at 0.1 cm in diameter. Other polypoidal tissues sent from the right middle meatus, left ethmoidal sinus, and septal masses were approximately 3×1 cm, and the cut section showed the cyst measured at 0.5 cm with pale brown to white surfaces suggesting the features of inflammatory polyps.

**Fig4:**
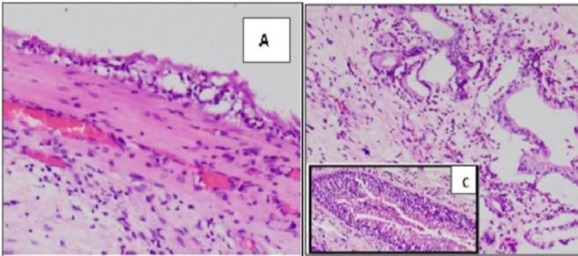
Microscopic picture (H and E stain); A) subepithelium with chronic inflammatory infiltrate with proliferating vessels (10×); B) hyperplastic seromucinous glands lined by cuboidal epithelium with focal areas of respiratory epithelium in a fibrous stroma ( 40×); C) magnified picture of respiratory epithelium-lined gland (100×)

The microscopic finding of the sections from polyp attached to the left middle turbinate showed polypoidal mucosa lined by respiratory epithelium with focal squamous metaplasia. Subepithelium showed edema and chronic inflammatory infiltrate. Hyperplastic seromucinous glands, as well as thick and thin proliferating vessels, were also noted. Many dilated glands lined by respiratory epithelium were observed. Some of the glands were distended with mucin and lined by compressed epithelium. Thick eosinophilic material with chronic inflammatory cells was observed condensed around the glands (Fig.4). The diagnosis was respiratory epithelial adenomatoid hamartoma (READ). The patient had no evidence of recurrent or residual disease during 6 months after the surgery.

## Discussion

Hamartoma was firstly coined in 1904 as tumor-like or primary, nonneoplastic malformations or in-born developmental errors of tissues (4). The READ is a subgroup of hamartoma described for the first time as a specific clinicopathological entity by Wenig and Heffner (1995) with the details of 31 patients (5). They are benign tumors that originate from epithelial surfaces with the components of glands stemming from these epithelia rather than the seromucinous glands.

Hamartomas are extremely rare lesions in the nose and sinuses. They are more common in the lungs, kidneys, liver, spleen, and intestines (1). Hamartomas occur due to the erroneous development of tissues and can arise from any epithelial surfaces, seromucous gland, fibrous stroma, or vessel (1,2). The hamartoma of the nose and sinuses is very rare, and its subtype READ was described only around 2 decades ago. Sinonasal hamartomas have been recently added and recognized as separate disease entity by the World Health Organization (6)*.*

Hamartomas cannot grow continuously by themselves; therefore, they have no malignant potential (5).

Although there has been more evidence regarding this entity since the first series of 31 cases of READ described by Wenig’s other details, such as etiopathogenesis and radiological, as well as clinical characteristic features are still not known (5). About 80% of cases with READ are men from the 3^rd^ to 9^th^ decade (with the median of 6^th^ decade) (1,3-10). No particular agents of etiology have been linked, including tobacco and alcohol consumption, that is similar to the findings of the present study (5). Patients present with nonspecific and vague symptoms, such as nose block, stuffy nose, epistaxis, running nose, and allergic symptoms (11). According to Wenig BM et al., hamartoma in the nose is commonly observed in the posterior septum (5). Endo R et al. described a case with a yellow mass in the left side of the nose touching the nasal floor and partial thinning of hard palate (1). 

Delbrouck C et al. described a case of READ in the left nasal cavity arising from behind the middle turbinate from the lateral wall and with nasal polyps in the ethmoid sinus (10). In addition, there are no specific findings in nasal endoscopy to confirm READ. Although preoperative imaging is essential there are not any specific findings in CT or magnetic resonance imaging to differentiate READ and other sinonasal diseases. 

The patient in this case report is an elderly male in the 7^th^ decade of life with hypertension presenting with nonspecific nasal symptoms with no history of long-standing sinusitis or allergic symptoms. In the evaluation through diagnostic nasal endoscopy, there were firm polypoidal masses in the lateral wall and septum anteriorly on both sides, while the masses described in previous studies arose from the posterior aspect of the septum. The results of CT scans of paranasal sinuses showed the features of chronic sinusitis and polypoidal changes in the bilateral middle meatus. During the surgery, these nasal masses were firm, pale, and polypoidal adherent to the surrounding structures. 

How hamartoma develops is still unknown. As READ is sometimes noticed with inflammatory polyps it is speculated that long-standing inflammation can be a cause for that (11). Histological changes mainly consist of proliferating gland-like structure. In areas, the glands arise directly from the epithelial surfaces invaginating into the submucosa. The glands are lined by multi-layered ciliated respiratory epithelium admixed with mucus-producing goblet cells. The hyalinization of stroma and glands enveloped by thick eosinophilic basement membrane are characteristically observed (12). In addition to glandular proliferation, there are extra findings associated with READ, including the alterations of the nasal polyp, hyperplastic and/or (squamous) metaplastic epithelial surfaces, metaplastic bone, inverted papilloma, and solitary fibrous tumor. 

On the contrary, molecular profile showing the presence of mean fractional allelic loss of 31% in READ leads to the suspicion that these lesions may represent benign neoplasias rather than hamartomas. On histology, important features are proliferating and accumulating glands and ducts lined by respiratory epithelium without atypical or metaplastic changes (1).

No particular immunohistochemistry staining is available for READ; however, MIB-1 staining may help differentiate hamartoma from neoplasms because neoplasms have more immunoreaction for MIB-1 than hamartomas (1). Differential diagnosis of significance includes sinonasal inflammatory polyp as READ may appear polypoidally. 

The fact that READ originates posteriorly from septum is in contrast with the polyps, which arise from the lateral walls of nose noted in the present case. Another differentiating feature of the polyps is that READ tends to be firmer than inflammatory polyps, which is referred by clinicians as being unusual for an inflammatory polyp. Other important differential diagnoses include inverted papilloma, nasopharyngeal angiofibroma, and adenocarcinoma among which the latter two need special attention as they warrant radical surgical intervention in contrast to the treatment of hamartoma that is completely excised locally (1-3). There is no evidence of recurrent, persistent or progressive diseases observed in previous studies up to the present (9).

## Conclusion

Hamartomas and more importantly its subtype READ are extremely rare lesions encountered in the sinonasal tract. Otorhinolaryngologists and pathologists should be aware of this entity as a differential diagnosis of inverted papilloma and adenocarcinoma to avoid misdiagnosing these lesions as actual neoplasia. Thereby subjecting patients to radical operations and misdiagnosing it as chronic sinusitis might lead to suboptimal therapy. The complete local excision of the lesions with histopathological confirmation is important to ensure adequate treatment.
